# Impact of a probiotic chewable tablet on stool habits and microbial profile in children with functional constipation: A randomized controlled clinical trial

**DOI:** 10.3389/fmicb.2022.985308

**Published:** 2022-08-22

**Authors:** Dan Gan, Jialun Chen, Xin Tang, Luyao Xiao, Christopher J. Martoni, Gregory Leyer, Guixia Huang, Wei Li

**Affiliations:** ^1^Sirio Pharma Co., Ltd., Shantou, China; ^2^College of Food Science and Technology, Nanjing Agricultural University, Nanjing, China; ^3^Chr. Hansen, Windsor, WI, United States

**Keywords:** functional constipation, microbiome, probiotic chewable, children, clinical trial

## Abstract

Constipation is a common and typically multifactorial childhood complaint, and the clinical management of childhood functional constipation (FC) is challenging. A randomized, single-blind, placebo-controlled, multi-center clinical trial was conducted in 92 children (47 from Beijing, China and 45 from Shanghai, China) aged 4–12 with FC according to Rome III criteria. Children were assigned to receive a probiotic chewable tablet (5 × 10^9^ CFU/day, *n* = 47), consisting of *Lactobacillus acidophilus* DDS-1^®^ and *Bifidobacterium animalis* subsp. *lactis* UABla-12™ or placebo (*n* = 45), twice daily for 4 weeks, followed by a week follow-up period. Results suggested that the probiotic group showed a faster and more pronounced normalization of stool frequency over the intervention period (3.15 vs. 1.83) when compared to placebo group (2.51 vs. 1.87). Meanwhile, the percentage of subjects with hard defecation decreased from 43 to 14% in the probiotic group, while the percentage of subjects with normal defecation increased from 56 to 80% in the probiotic group, further confirming the normalization of stools habits. This randomized controlled trial demonstrated the potential of a probiotic chewable tablet containing *L. acidophilus* DDS-1^®^ and *B. Lactis* UABla-12™ as a daily probiotic dosage form for children with FC.

## Introduction

The global prevalence of functional constipation (FC) is approximately 10–15%, with higher prevalence rates in women and older individuals (Zhao et al., 2011; Barberio et al., 2021). The gut microbiota refers to the microbial community present in the human gastrointestinal tract, consisting of trillions of bacteria, viruses, fungi, archaea, and eukaryotes. Although there is no standard definition of a healthy gut microbiota, it is generally characterized by a microbiota with high levels of diversity, stability, resistance to stress-related changes, and high levels of redundancy in metabolic pathways (Bäckhed et al., 2012).

The intestinal microbiota is mutually constrained and interdependent to maintain a certain ecological balance. Further, it helps maintain the structural integrity of the intestinal mucosal barrier (Jandhyala et al., 2015), and participates in several physiological processes in the host. Probiotics are defined as “live microorganisms that, when administered in adequate amounts, confer a health benefit on the host” (Hill et al., 2014; Salminen et al., 2021). Probiotics have been suggested to beneficially modulate FC through a variety of potential mechanisms. First, they may help support the intestinal microenvironment by producing organic acids, such as lactic and acetic acid, which lowers intestinal lumen pH and may stimulate intestinal motility (Zoppi et al., 1998). Additionally, certain probiotic strains have been reported to shorten colonic transit time (Marteau et al., 2002; Waller et al., 2011). The intestinal microbiota not only regulates intestinal activity, but also influences and regulates host brain function and behavior through the gut-brain axis (Tillisch et al., 2013; Ghouri et al., 2014). In a prior study, a probiotic blend helped modulate visceral sensory and somatosensory cortical areas of the brain and had significant and characteristic effects on intestinal epithelial cells, intestinal immune function and the enteric nervous system (Khalif et al., 2005).

It has been shown that adults with constipation exhibit a decrease in *Bifidobacterium* spp. and *Lactobacillus* spp. and an increase in potentially pathogenic bacteria and fungi (Huang et al., 2018). At present, the treatment options for FC may include oral medications, enemas, biofeedback, and surgical treatment. Laxatives and prokinetics are often the first-line treatment option, but they are for short term use and have a number of potential side effects. Alternatively, probiotics and prebiotics are widely administered in the treatment of FC because of their safety and potential efficacy (Tannock, 2004). There have been several studies on probiotics for FC, most of which have focused on observing efficacy through improvements in stool frequency, stool properties, and clinical symptoms associated with constipation (Dimidi et al., 2014).

A total of 1,182 adult FC patients were included in a meta-analysis showing that probiotics reduced bowel motility time by 12 h/week and increased stool frequency by 1.3 times/week, and were associated with the type of probiotic, with *Bifidobacterium bifidum* significantly improving symptoms and *Lactobacillus casei* Shirota having no benefit (Dimidi et al., 2014). Botelho et al. (2020) found that multispecies probiotics in capsule form may modulate the balance of gut microbiota by reducing the relative abundance of bacteria in patients with constipation based on a 30-days clinical trial. Functional constipation is a common childhood complaint and is typically multifactorial. Clinical management of childhood FC is challenging and more studies examining the utility of probiotics are warranted. As such, the aim of the current study was to assess the efficacy of a probiotic chewable in children with FC (Chmielewska et al., 2011; Wang et al., 2017).

## Population and methods

### Study population

The study was conducted in accordance with the ethical principles that have their origins in the Declaration of Helsinki and its subsequent amendments. The clinical trial was registered on clinicaltrials.gov under registration number ChiCTR2000038603, and was conducted according to the CONSORT 2010 Statement.

Children, aged 4–12 years with FC were enrolled. The included participants met the requirements of Rome III criteria for FC based on self-reporting over the past 3 months, with symptom onset beginning within the previous 6 months. Additionally, participants were required to have an average stool type of <3 on the Bristol stool scale (BSS), as assessed over a 2-week run-in period and agreed to maintain their current level of physical activity throughout the trial period. Exclusion criteria included type I or type II diabetes, cancer, neurological disorders, immunocompromised conditions, major diseases of the cardiovascular, renal, hepatic, gastrointestinal, pulmonary or endocrine systems, a history of gastrointestinal complications (such as inflammatory bowel disease and ulcers) or abdominal surgery, a history of heavy drinking or an allergy or sensitivity to the test product ingredient. The use of antibiotics, probiotics, fiber supplements or prebiotic fiber/enriched foods were prohibited within 4 weeks prior to screening and during the trial. All participants provided their voluntary, written, informed consent prior to their inclusion.

### Study design

A randomized, single-blind, placebo-controlled, multi-center clinical trial research design was used in this study for 42 days. The trial was divided into four periods, observation period (1 week), intervention period 1 (weeks 1 and 2), intervention period 2 (weeks 3 and 4), and follow-up period (1 week). After completing the first week of testing, all children’s feedback data were verified, based primarily on pictures of bowel movements and the number of assisted bowel movements. After confirmation of eligibility, probiotic chewable tablets and placebo were distributed for follow-up testing. No intervention was performed during the observation period. During the intervention period, a chewable probiotic tablet or placebo was administered twice a day, once in the morning and once in the evening, for 4 weeks. Weekly stool frequency was recorded, stool morphology was evaluated via BSS, stool pictures were kept, and stool samples were collected from the subjects for extraction of total DNA for high-throughput biological analysis of 16S bacteria. The names of the 16S ribosomal deoxyribonucleic acid (rDNA) sequencing groups are shown in Table 1.

**TABLE 1:** Name of 16S rDNA sequencing groups.

Group	Intervention content	Intervention time	Abbreviations
Treatment 1	None	1 week	A0
Treatment 2	Probiotic chewable tablet	2 weeks	A1
Treatment 3	Probiotic chewable tablet	4 weeks	A2
Control 1	None	1 week	B0
Control 2	Placebo	2 weeks	B1
Control 3	Placebo	4 weeks	B2

### Study product

The probiotic chewable tablets contained two strains: *L. acidophilus* DDS-1^®^ and *Bifidobacterium animalis* subsp. *lactis* UABla-12™. The probiotic chewable tablets, which each contained not less than 5 × 10^9^ CFU/tablet, were formulated with lyophilized probiotic blend, 1% hydroxymethyl cellulose and 1% magnesium stearate, and prepared by a tablet press. Placebo chewable tablets did not contain probiotic blend, but otherwise were identical in mass, taste, appearance and odor. Both probiotic and placebo chewable tablets met all quality specifications, including potency and bacterial culture purity, microbiological analysis, color, appearance, weight specifications/variation and disintegration at the time of manufacture and at the end of the clinical study.

### Outcome assessments

Stool frequency was recorded and stool morphology was assessed, via the BSS, throughout the 4-week intervention period. Stool samples were collected with the help of a parent or guardian. Children defecated on test strips to avoid contact of stool with urine. Stool samples were collected with sampling tubes and immediately placed in a 4°C environment for storage and transported frozen throughout until completion of 16S rDNA sequencing.

### Microbial profiling analysis

DNA was extracted from fecal samples and the quality of the extracted genomic DNA was examined by 1% agarose gel electrophoresis. DNA was subsequently amplified using fusion primers for the 16S v3v4 region, and specific primers with 5′ barcode were synthesized. The PCR amplification products were purified and recovered from magnetic beads, quantified by Quant-iT PicoGreen dsDNA Assay Kit, and the samples were mixed in the appropriate ratio according to the quantification results. Sequencing libraries were prepared by TruSeq DNA HT Sample Prep Kit, and the final fragments of the libraries were optimized by 2% agarose gel electrophoresis. MiSeq Reagent Kit V3 was used for sequencing. The raw data were filtered to optimize the sequences. The resulting sequences were compared with the Greengenes database, the sequences with >97% similarity were assigned to the same operational taxonomic unit (OTU), and strain abundance was calculated using random sampling of sequenced sequences, and dilution curves were constructed using the number of sequences sampled versus the number of OTUs they could represent, and Wilcoxon signed-rank test for alpha diversity analysis, Principal coordinates analysis (PCoA), phylum level and genus level analysis. The sequence data have been deposited in the Sequence Read Archive (SRA) database at the National Center for Biotechnology Information (NCBI) under accession number PRJNA862261.

### Statistical analysis

GraphPad Prism 8.0 Software was used for the statistical analysis of stool frequency and BSS value. Data are presented as the mean ± standard deviation (SD). *Student’s t*-test was used for comparisons between two groups. One-way analysis of variance (ANOVA) followed by *Tukey’s post-hoc* test was used to compare differences among three or more groups. Results of 16SrDNA sequencing used *Wilcoxon signed-rank* to test the significance of differences. Values of *p* < 0.05 were considered statistically significant.

## Results

### Study parameters

**FIGURE 1:**
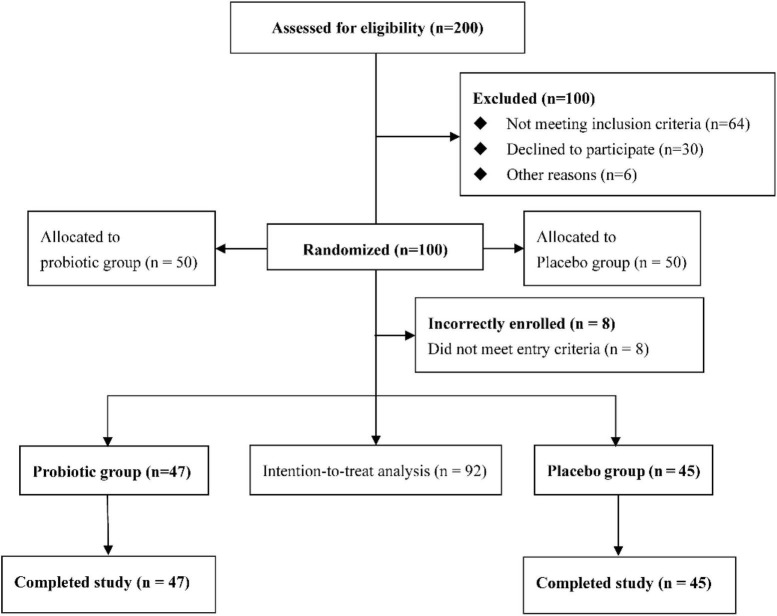
Participants’ enrollment.

A total of 100 children were enrolled. Of these, three children from the probiotic group in Beijing had incomplete data, and five children from the placebo group in Shanghai had incomplete data. Ninety-two children completed the study, with 47 children in the probiotic group (22 from Beijing and 25 from Shanghai), and 45 children in the placebo group (25 from Beijing and 20 from Shanghai) the sex radio for probiotic group and placebo group was 23:24 and 23:22, respectively. Meanwhile, the average age was 8.4 years in the probiotic group and 8.1 years in the placebo group (Figure 1).

### Stool habits

**FIGURE 2:**
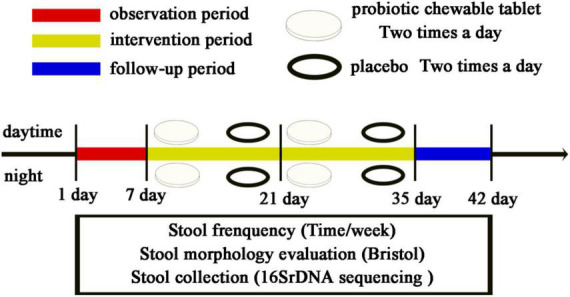
Study design.

**FIGURE 3:**
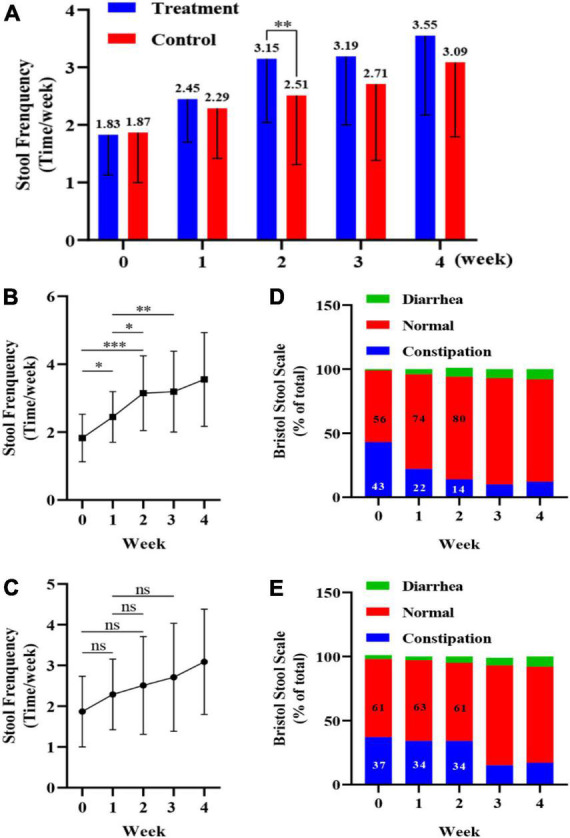
Impact of a probiotic chewable tablet on stool habits. **(A)** Stool frequency during the observation period and after 4 weeks of intervention. ^**^p < 0.01, *Student’s t*-test. **(B)** Change in stool frequency during the observation period and intervention period for children in the probiotic (treatment) group. *p < 0.05, ^**^p < 0.01, ^***^p < 0.001, *Tukey’s post-hoc* test. **(C)** Change in stool frequency during the observation period and dry period in children in the placebo (control) group. ^ns^*p* > 0.05, *Tukey’s post-hoc* test. **(D)** Percentage of stool type, via the Bristol stool scale (BSS), in the treatment group during the observation and intervention periods. **(E)** Percentage of stool type, via the BSS, in the control group during the observation and intervention periods.

The study design is shown in Figure 2. During the 7-day observation period (0-week), all children had fewer than two bowel movements per week, and there was no significant difference between the treatment and control groups. At 2 weeks, the number of bowel movements per week increased significantly in the probiotic group (*p* < 0.01) compared to the placebo group, with an average number of bowel movements of greater than three per week. After 4 weeks the average number of bowel movements per week was 3.55 in the probiotic group, corresponding to one bowel movement every 2 days (Figure 3A). Among the children in the probiotic group, stool frequency was significantly higher after week 1 (*p* < 0.05) and week 2 (*p* < 0.001) compared to week 0. In contrast, there was no change in stool frequency after weeks 1 and 2, as compared to week 0, among the children receiving placebo (*p* > 0.05). In addition, stool frequency increased significantly in the probiotic group at both week 2 and week 3, as compared to week 1 (*p* < 0.05, Figure 3B). No significant difference was observed in stool frequency over the same timepoints in the placebo group (*p* > 0.05; Figure 3C).

Stool morphology in children was determined via the BSS, with scores of 1 or 2 and 6 or 7 corresponding to constipation and diarrhea, respectively, and scores from 3 to 5 corresponding to normal stools. For each child, the ratio of the number of occurrences (i.e., constipation, normal, and diarrhea) to the total number of stools (%) was calculated, and the results are shown in Figures 3D,E. From week 0 to week 2, the percentage of normal stools increased from 56 to 80% in the probiotic group, while the percentage of constipated stools decreased from 43 to 14% (Figure 3D). In the placebo group the percentage of normal stools was 61% at both weeks 0 and 2, while the percentage of constipated stools was 37 and 34% at week 0 and 2, respectively (Figure 3E).

### Microbial profiling

#### Alpha diversity analysis

**FIGURE 4:**
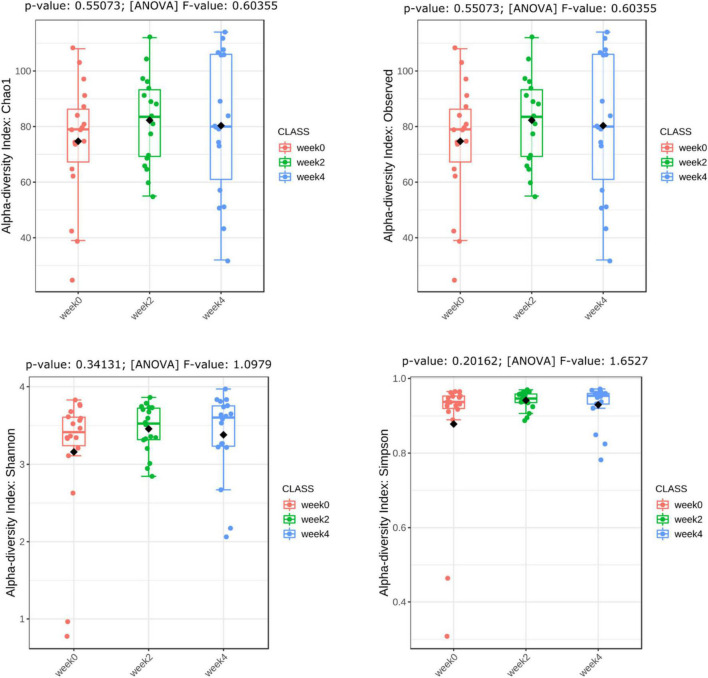
Alpha diversity estimates in probiotic group.

This procedure characterizes richness by Chao1 and Observed indices and diversity by Shannon and Simpson indices. The alpha diversity of the treatment and placebo groups were examined after the observation period (week 0), the intervention period of 2 weeks (week 2) and the intervention period of 4 weeks (week 4), respectively. In the probiotic group, there were no significant differences in strain richness or diversity at weeks 2 and 4 as compared to week 0 (Figure 4).

#### Beta diversity analysis

**FIGURE 5:**
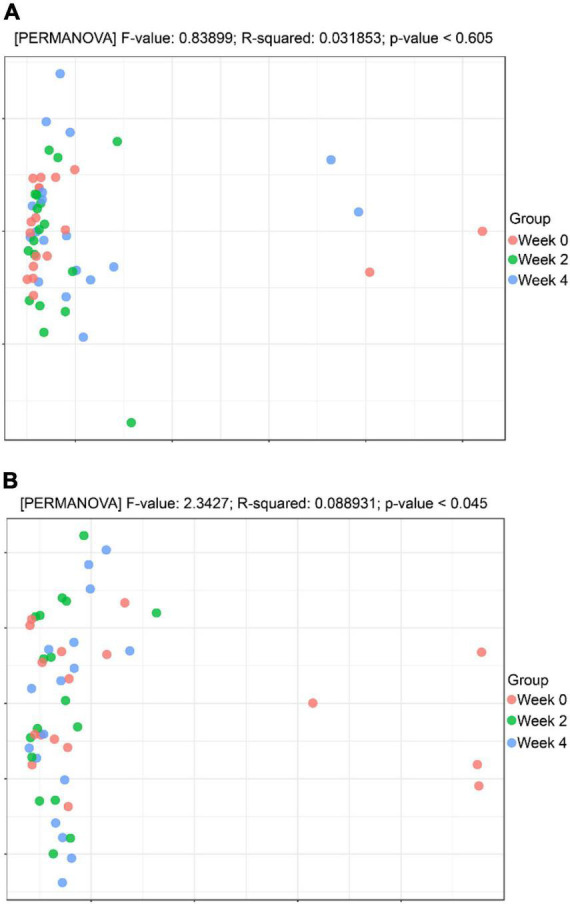
Beta diversity analysis. **(A)** Principal coordinates analysis (PCoA) analysis in the probiotic (treatment) group. **(B)** PCoA analysis in the placebo (control) group.

This procedure was used to observe the differences between individuals or groups by weighted UniFrac based PCoA and Permanova method to test the significant differences between sample groups. As shown in Figure 5A, in the probiotic group, there was less variability when comparing weeks 0, 2, and 4 (*p* = 0.969) as compared to the placebo group, which showed more variability when comparing weeks 0, 2, and 4 (*p* = 0.045; Figure 5B).

#### Microbial community analysis

**FIGURE 6:**
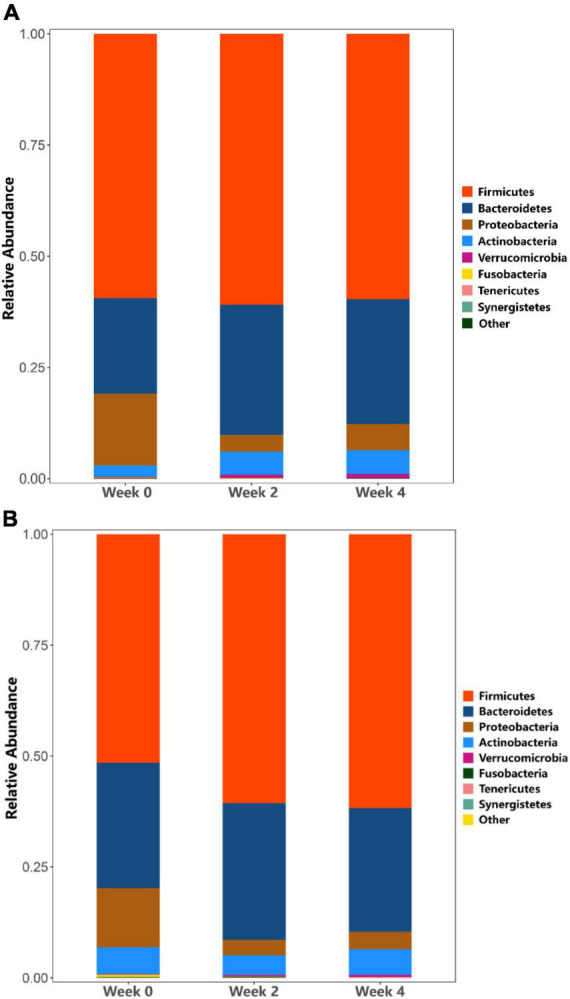
Analysis of phylum taxa abundance. **(A)** Phylum taxa abundance in the probiotic (treatment) group. **(B)** Phylum taxa abundance in the placebo (control) group.

**FIGURE 7:**
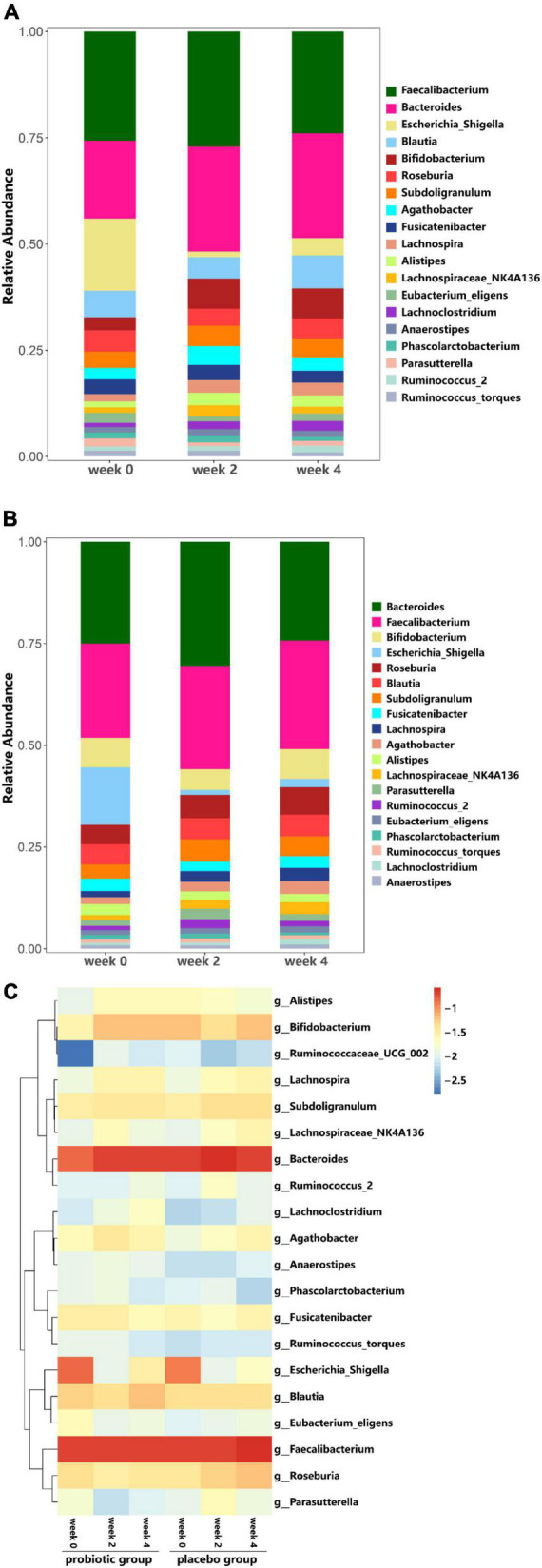
Analysis of Family and Genus taxa abundance. **(A)** Genus taxa abundance in the probiotic group. **(B)** Genus taxa abundance in the placebo group. **(C)** Heat map of top 20 genera.

At the phylum level, in probiotic group, the relative abundance of Actinobacteria (*Bifidobacteria*) was higher at weeks 2 and 4 as compared to week 0, while the relative abundance of *Proteobacteria* decreased (Figure 6A). In placebo group, the relative abundance of Actinobacteria (*Bifidobacteria*) remained unchanged throughout the study period, while the relative abundance of Proteobacteria decreased (Figure 6B). Additionally, the probiotic group exhibited higher relative abundance of *Clostridiaceae* (*p* = 0.011), *Streptococcaceae* (*p* = 0.031), and *Peptostreptococcaceae* (*p* = 0.039) at week 4 compared to week 0, however, these differences were not significant when accounting for multiple comparisons (Figures 7A,B). Figure 7C shows a heat map of the top 20 genera, with the color gradient from blue to red representing change from less to more. In the probiotic group, the relative abundance of *Bifidobacteria* increased after 2 and 4 weeks of intervention compared to week 0. Interestingly, after 2 and 4 weeks of intervention, the relative abundance of *Escherichia-Shigella* decreased in both groups.

## Discussion

The present study was a randomized placebo-controlled trial to assess a probiotic chewable tablet consisting of *L. acidophilus L. acidophilus* DDS-1^®^ and *B. Lactis* UABla-12™ in children with FC living in Shanghai or Beijing. Previously, the two strains, alone or in combination, were shown to improve abdominal pain severity and symptomology in irritable bowel syndrome, support abdominal symptom relief for lactose intolerance, reduce the severity of atopic dermatitis and reduce the severity and duration of acute viral respiratory tract infection in randomized controlled trials (Martoni et al., 2020). Consistent with previous studies, probiotic treatment led to changes in constipation symptoms in children meeting Rome III criteria for FC (Drossman et al., 2009; Barish et al., 2010). Previously, a cluster analysis and diversity analysis were performed on sigmoid mucosal microflora and stool samples of constipated patients, respectively, and found that changes in sigmoid mucosal microflora were more closely related to constipation (Parthasarathy et al., 2016). Therefore, it has been suggested that standardizing the method of measuring intestinal flora may help in the assessment of intestinal microbiota changes in FC.

The most immediate sign of improvement in constipation is a change in stool frequency and bowel sensation. In the current study, children in the probiotic group showed a linear increase in stool frequency after the first and second weeks (Figure 3B). By the second week of treatment, children in the probiotic group showed a clinically relevant effect, with an average of 3.15 bowel movements per week, which was significantly greater than that of the placebo group. This was also in line with a meta-analysis of probiotic studies in adults that demonstrated increased stool frequency, by an average of 1.3 bowel movements per week, compared with a placebo. It is noteworthy that stool consistency, via BSS, showed a similar improvement over the study period. More specifically, the percentage of constipation-type stools in the 2 weeks after probiotic treatment decreased from 43 to 14%. Combining stool frequency and consistency, the most noticeable improvement in the probiotic group was observed over the first 2 weeks, followed by a diminishing effect vs. placebo after the third week.

Previous studies have reported a correlation between certain gut microbes and constipation, particularly in terms of transit time (Bouvier et al., 2001; Khalif et al., 2005; Jandhyala et al., 2015). Our sequencing results showed that in the probiotic group, there was no significant difference in strain richness or diversity over the study period (Figure 4), regardless of their stool frequency. The α and β diversity did not differ between the three intervention time periods in the probiotic group (Figures 4, 5A, respectively). Interestingly, we observed an increase in alpha diversity in the stools of the placebo group (Figure 4), with a PCoA analysis revealing a difference between and weeks 2 and 4 as compared to week 0. The significance of this increase is uncertain, as an increase in microbial diversity may be considered beneficial for human gut health (De Filippo et al., 2010; Qin et al., 2010; Kang et al., 2013). In the context of this study, a larger number of participants would likely be needed to better understand this observation. We did not observe significant differences in microbial profiling of participants with constipation vs. normal stool profiles. Previous studies have reported either higher or lower levels of *bifidobacteria* in stool samples from constipated patients compared to healthy subjects (Khalif et al., 2005). In the current study, the probiotic group showed an increase in the relative abundance of Actinobacteria (*Bifidobacteria*), while a decrease in the relative abundance of *Proteobacteria* (Figure 6A). In the placebo group, the relative abundance of Actinobacteria (*Bifidobacteria*) remained unchanged, while the relative abundance of Proteobacteria decreased (Figure 6B). Fecal samples of the probiotic group exhibited higher relative abundance of *Clostridiaceae*, *Streptococcaceae*, and *Peptostreptococcaceae* at endpoint compared with baseline, however, these differences were not significant when accounting for multiple comparisons (Figure 7A). Previously, it has been reported that lactobacilli levels were significantly lower in adults with constipation as compared to healthy controls (Khalif et al., 2005). However, lactobacilli levels were similar when comparing children with and without constipation (Zhu et al., 2014; Jandhyala et al., 2015). Potentially, differences between studies may be attributed to the techniques used to identify microorganisms and in the difficulty of simulating the gastrointestinal environment (Hugenholtz, 2002).

Khalif et al. (2005) observed few differences in the gut microbiota between healthy and constipated individuals, unless constipated subjects with severely prolonged transit times were considered in the comparison. Symptom-based diagnoses, such as the Rome III criteria, may not be sufficient to distinguish between normal and constipated subjects regarding differences in gut microbiota. However, the Bristol stool score has been shown to correlate well with transit time, and we did not observe differences based on this parameter. Using delayed/difficult defecation, symptom frequency, and painfulness as definitions of constipation, Zhu et al. (2014) found differences in beta microbial diversity between obese children with and without constipation. Increasing studies have demonstrated that the occurrence of FC is closely associated with the imbalances of gut microbiota (Cao et al., 2017; Ohkusa et al., 2019). Meanwhile, the deficient motor-propulsive pattern of the intestine seems to be an important cause of constipation. It has been confirmed that gut microbiota can affect intestinal motility directly, through the release of metabolic products such as short-chain fatty acids (SCFAs), or indirectly through the regulation of the immune system (Dimidi et al., 2017; Su et al., 2020; Yang et al., 2021). Thus, the reconstruction of intestinal microbial community and the improvement of intestinal motility would be effective in alleviating constipation symptoms.

## Conclusion

In summary, this randomized controlled trial assessed a probiotic chewable containing *L. acidophilus* DDS-1^®^ and *B. animalis* subsp. *lactis* UABla-12™ for its effect on bowel habits and microbial profile simultaneously. The product was well tolerated and its effects on stool frequency and consistency indicate a potential benefit as a daily probiotic dosage form for children with FC.

## Data availability statement

The data that support the findings of this study are openly available in the Sequence Read Archive (SRA) database at the NCBI under accession number PRJNA862261.

## Ethics statement

The studies involving human participants were reviewed and approved by China Ethics Committee of Registering Clinical Trials. Written informed consent to participate in this study was provided by the participants’ legal guardian/next of kin.

## Author contributions

DG, JC, XT, and WL: conceptualization. DG, JC, XT, and CM: methodology. DG and XT: statistical analysis and the analysis of the results. DG, JC, XT, and LX: writing—original draft preparation. GL, GH, and WL: writing—review and editing. All authors have read and agreed to the published version of the manuscript.
